# Adaptive Multimodal Fusion in Vertical Federated Learning for Decentralized Glaucoma Screening

**DOI:** 10.3390/brainsci15090990

**Published:** 2025-09-14

**Authors:** Ayesha Jabbar, Jianjun Huang, Muhammad Kashif Jabbar, Asad Ali

**Affiliations:** 1 College of Electronics and Information Engineering, Shenzhen University, Shenzhen 518060, China; ayeshajabbar2023@email.szu.edu.cn (A.J.); muhammadkashifjabbar@email.szu.edu.cn (M.K.J.); aliasad2023@email.szu.edu.cn (A.A.); 2 Guangdong Provincial Key Laboratory of Intelligent Information Processing, Shenzhen University, Shenzhen 518060, China

**Keywords:** vertical, federated learning, multimodal fusion, glaucoma detection, privacy-preserving healthcare AI, attention-based neural networks

## Abstract

**Background/Objectives:** Early and accurate detection of glaucoma is vital for preventing irreversible vision loss, yet traditional diagnostic approaches relying solely on unimodal retinal imaging are limited by data sparsity and constrained context. Furthermore, real-world clinical data are often fragmented across institutions under strict privacy regulations, posing significant challenges for centralized machine learning methods. **Methods:** To address these barriers, this study proposes a novel Quality Aware Vertical Federated Learning (QAVFL) framework for decentralized multimodal glaucoma detection. The proposed system dynamically integrates clinical text, retinal fundus images, and biomedical signal data through modality-specific encoders, followed by a Fusion Attention Module (FAM) that adaptively weighs the reliability and contribution of each modality. Unlike conventional early fusion or horizontal federated learning methods, QAVFL operates in vertically partitioned environments and employs secure aggregation mechanisms incorporating homomorphic encryption and differential privacy to preserve patient confidentiality. **Results:** Extensive experiments conducted under heterogeneous non-IID settings demonstrate that QAVFL achieves an accuracy of 98.6%, a recall of 98.6%, an F1-score of 97.0%, and an AUC of 0.992, outperforming unimodal and early fusion baselines with statistically significant improvements (*p* < 0.01). **Conclusions:** The findings validate the effectiveness of dynamic multimodal fusion under privacy-preserving decentralized learning and highlight the scalability and clinical applicability of QAVFL for robust glaucoma screening across fragmented healthcare environments.

## 1. Introduction

The rise of data-driven technologies in healthcare has opened transformative opportunities for early disease diagnosis, risk stratification, and personalized treatment planning [1]. Among these opportunities, machine learning models that integrate multimodal clinical data have shown significant promise in improving diagnostic accuracy beyond what single modality systems can achieve [2]. Multimodal data sources ranging from imaging studies and textual clinical narratives to physiological time series signals offer rich, complementary information that, when fused appropriately, can provide a holistic and nuanced understanding of patient conditions, disease progression, and therapeutic outcomes [3]. In the context of glaucoma detection, early and accurate diagnosis is crucial to prevent irreversible vision loss, which often remains asymptomatic until significant damage has occurred [4]. Although retinal fundus imaging remains the primary diagnostic modality, recent research has highlighted the critical value of integrating additional sources of clinical information such as intraocular pressure measurements, visual field tests, optical coherence tomography (OCT) data, clinical notes, and biosensor readings to enhance diagnostic certainty and robustness [5]. The synergy of these modalities offers a more comprehensive patient profile, potentially enabling earlier detection of pathological changes that are not evident through imaging alone [6].

**Figure 1:**
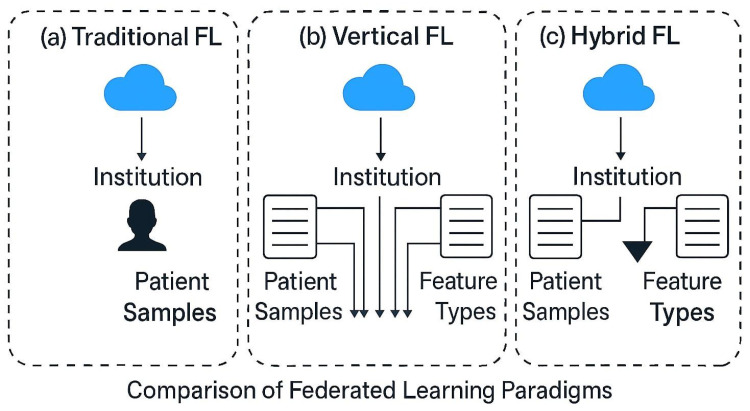
Comparison of Federated Learning Paradigms. (**a**) Traditional centralized learning: patient data from all sites are pooled into a single repository for training. (**b**) Vertical Federated Learning (our case) distributes complementary feature types (modalities) across institutions. (**c**) Hybrid FL combines both horizontal and vertical paradigms.

However, building machine learning systems that effectively leverage such diverse modalities presents multiple challenges. One major hurdle is the imbalance of modes, where certain modalities may be missing, corrupted, or available only for a subset of patients [7]. Another critical issue is fragmentation between institutions, where different healthcare providers have partial information about the same patient due to the distributed nature of healthcare delivery [8]. Furthermore, stringent privacy regulations, such as the Health Insurance Portability and Accountability Act (HIPAA) in the United States and the General Data Protection Regulation (GDPR) in Europe, severely restrict the centralized pooling of patient data, posing significant barriers to traditional machine learning approaches that rely on aggregating all data in a single location [9]. Federated learning (FL) emerges as a compelling paradigm to address these challenges by enabling the training of a decentralized model among distributed data holders without requiring the transfer of sensitive raw data, as illustrated in Figure 1.

However, building machine learning systems that effectively leverage such diverse modalities presents multiple challenges. One major hurdle is modality imbalance, where certain modalities may be missing, corrupted, or available only for a subset of patients [10]. Another critical issue is fragmentation across institutions, where different healthcare providers possess partial information about the same patient due to the distributed nature of healthcare delivery [8]. Furthermore, stringent privacy regulations, such as HIPAA in the United States and GDPR in Europe, severely restrict the centralized pooling of patient data, posing significant barriers to traditional machine learning approaches that rely on aggregating all data in a single location. FL emerges as a compelling paradigm to address these challenges by enabling decentralized model training across distributed data holders without requiring the transfer of sensitive raw data. Nonetheless, the majority of current FL methods operate under the assumption of horizontal partitioning, where different clients hold data samples with identical feature spaces but distinct patient populations [11]. In contrast, real-world clinical datasets often exhibit vertical partitioning, wherein different institutions possess different modalities or feature sets for the same group of patients [12]. Such settings necessitate specialized federated frameworks capable of learning jointly from distributed, feature-complementary data without compromising privacy [13].

Moreover, naive modality fusion strategies, such as simple feature concatenation or early fusion techniques, are ill suited for real-world multimodal clinical datasets [8]. These datasets often exhibit substantial heterogeneity in data quality, missingness patterns, sampling rates, and statistical distributions across modalities. Consequently, a more sophisticated and dynamic fusion mechanism is essential, capable of adaptively weighting the reliability and contribution of each modality during both training and inference to maximize diagnostic performance [14]. This paper addresses these critical gaps by proposing a QAVFL framework specifically designed for decentralized multimodal glaucoma detection [15]. The proposed method introduces a novel Fusion Attention Module (FAM), which dynamically attends to the quality and presence of each modality, enabling selective feature integration that is robust to modality imbalance and missing data scenarios [16]. In parallel, secure aggregation mechanisms and encrypted communication protocols are incorporated to ensure that patient confidentiality is strictly preserved throughout the federated training process [17]. By leveraging QAVFL, we aim to enable privacy-preserving, cross-institutional collaborative learning that significantly enhances the accuracy and reliability of early glaucoma detection in heterogeneous, real-world clinical environments.

### 1.1. Problem Statement

Glaucoma diagnosis increasingly benefits from multimodal clinical data; however, the fragmentation of patient information across different institutions and the presence of strict data privacy regulations severely limit the feasibility of centralized learning approaches. Existing FL solutions predominantly focus on horizontally partitioned data and are insufficient for vertically partitioned settings where different modalities are siloed across clients. Furthermore, current modality fusion techniques lack adaptability, failing to handle missing, imbalanced, or noisy modalities effectively, thus degrading model robustness and diagnostic reliability. A critical need exists for a privacy-preserving, vertically federated framework capable of dynamically attending to the quality and availability of multimodal clinical data, enabling robust decentralized learning without compromising patient privacy.

### 1.2. Research Objectives

This research aims to develop a robust, privacy-preserving, and multimodal framework for decentralized glaucoma detection using vertical FL. The key objectives of this study are as follows:

- To design a scalable FL framework capable of handling vertically partitioned multimodal clinical data including retinal images, textual records, and biosignals distributed across ten distinct client nodes.
- To implement secure model communication and aggregation protocols using additive homomorphic encryption (AHE) and differential privacy (DP), ensuring that raw patient data remains locally protected at all times.
- To optimize local training on client nodes using the VGG16 backbone, Adam optimizer, and learning rate scheduling, with special handling of class imbalance through SMOTETomek integration.
- To construct a central server-side aggregation pipeline that fuses local model updates, aggregates gradients, and generates a global model capable of generalizing across non-IID clients and inconsistent modalities.
- To validate the effectiveness of the proposed system by evaluating it under realistic decentralized conditions using comprehensive metrics such as accuracy, precision, recall, F1-score, and AUC, while ensuring client-specific performance calibration.
- To demonstrate that the proposed solution offers a secure, interpretable, and scalable approach for clinical decision support, applicable across real-world healthcare institutions with heterogeneous data landscapes.

## 2. Related Work

Recent advances in machine learning for healthcare have demonstrated the transformative potential of multimodal data integration for improving diagnostic performance, patient stratification, and individualized care planning. However, existing approaches generally fall into three broad categories: unimodal models, early fusion multimodal models, and decentralized FL systems [18]. Each of these paradigms, while offering meaningful contributions, faces distinct challenges when confronted with the realities of clinical settings, where data heterogeneity, missing modalities, privacy regulations, and institutional data silos are prevalent [19].

Traditional machine learning and deep learning models for glaucoma detection have primarily focused on unimodal analysis, particularly using retinal fundus images. Convolutional neural networks (CNNs) such as ResNet, DenseNet, and VGG architectures have achieved impressive accuracy levels in identifying glaucomatous features, especially when trained on large, curated, and high-quality datasets [18]. However, these unimodal systems inherently neglect valuable complementary clinical information, including intraocular pressure (IOP) readings, corneal thickness measurements, visual field test results, family history, and physician notes [20]. As a result, unimodal approaches are often brittle and prone to significant performance degradation when faced with noisy, incomplete, or atypical image data, highlighting the critical need for richer, multimodal representations to achieve more robust and generalizable diagnostic systems [21].

In response to the limitations of unimodal systems, early fusion multimodal learning strategies have gained popularity [22]. These approaches aim to combine data from multiple modalities either at the raw input stage (early fusion) or at intermediate feature representation layers (mid-level fusion) [23]. Several studies have combined imaging modalities with electronic health record (EHR) data, biosignals, or genetic information to enhance disease classification and prognosis prediction [24]. For instance, one study successfully integrated dermatological images with patient history metadata to improve skin cancer diagnosis, while another study combined clinical notes with laboratory results for mortality prediction tasks [25]. Despite moderate improvements in performance, early fusion models often fail to account for real-world data imperfections [26]. They typically assume that all modalities are consistently available and of equal quality, an assumption that is rarely valid in clinical practice. In heterogeneous datasets, dominant modalities such as imaging may overpower weaker but still informative modalities, leading to biased models that are overly dependent on particular data types. Furthermore, simple concatenation of features does not accommodate cases where modalities are missing or partially available, making these systems brittle and vulnerable to missing data scenarios commonly encountered in healthcare [27].

To address data sharing constraints, FL has emerged as a promising decentralized machine learning paradigm, enabling multiple institutions to collaboratively train models without sharing raw patient data [28]. Initial applications of FL in healthcare predominantly focused on horizontally partitioned datasets, where different institutions contribute patient data samples that share the same feature space but represent disjoint patient cohorts. Landmark studies in brain tumor segmentation and in COVID-19 diagnosis have demonstrated that horizontally federated models can achieve performance comparable to traditionally centralized training while adhering to data privacy requirements [28]. Nevertheless, horizontal FL frameworks are inherently limited when applied to scenarios where different modalities or feature subsets of the same patients are distributed across institutions, which is increasingly common in distributed healthcare systems involving specialist clinics, hospitals, and laboratories [29].

Vertical FL (VFL) offers a more suitable framework for healthcare settings characterized by vertically partitioned data, where different institutions hold complementary features for the same patient population. VFL has been explored in domains such as EHR mining, genomics, and cross-institutional predictive modeling, with notable frameworks like SecureBoost and FedEHR leading early investigations [17]. However, most existing VFL methods simply concatenate encrypted features across institutions and perform model training without dynamically considering the reliability, relevance, or availability of each modality. This naive fusion approach inherits the brittleness of early fusion methods and exacerbates challenges under heterogeneous and incomplete data settings. Moreover, although privacy-preserving techniques such as homomorphic encryption, secure multiparty computation (SMPC), and differential privacy have been incorporated into some VFL systems, their integration often introduces significant computational overhead without adequately addressing the resulting trade-offs between privacy, communication efficiency, and model performance [30].

In addition to these technical limitations, current FL frameworks frequently neglect the substantial heterogeneity present in clinical data distributions across different institutions [31]. Variations in imaging protocols, diagnostic equipment, patient demographics, and data curation standards can cause significant domain shifts that undermine the performance of naively trained federated models [32]. Furthermore, clinical data are rarely complete; missing modalities, corrupted features, and partial information are ubiquitous challenges that simple concatenation or static fusion methods cannot effectively mitigate. The absence of adaptive fusion mechanisms that dynamically adjust to modality availability and reliability during training remains a significant shortcoming in current FL literature [33].

These important gaps motivate the development of the proposed QAVFL framework for decentralized multimodal glaucoma detection. Our approach introduces a novel FAM that dynamically integrates multimodal features by learning to attend to the most reliable and informative modalities at each training iteration. Unlike traditional static fusion schemes, FAM enables the model to flexibly handle missing modalities, varying data quality, and inconsistent modality contributions across different institutions. Furthermore, QAVFL employs secure aggregation protocols that ensure strict patient confidentiality throughout the training process without incurring prohibitive computational overhead. By addressing the limitations of unimodal systems, brittle early fusion strategies, and rigid FL architectures, QAVFL offers a scalable, privacy-preserving, and clinically robust solution for real-world decentralized glaucoma diagnosis [34].

## 3. Methodology

The proposed methodology introduces a novel framework termed QAVFL, specifically developed for decentralized glaucoma detection using vertically partitioned multimodal clinical data. In real-world healthcare settings, patient information is often scattered across multiple institutions, each retaining distinct modalities such as retinal fundus images, clinical notes, or biomedical signals. To collaboratively train a robust glaucoma classifier without compromising data privacy, QAVFL adopts an FL paradigm that prohibits raw data sharing. Each of the ten client nodes processes a unique modality via modality-specific encoders: clinical text is vectorized using TF-IDF and Word2Vec, retinal images are fed through a ResNet-18 backbone followed by Global Average Pooling (GAP), and biomedical signals are preprocessed using Savitzky Golay filtering and passed into a one-dimensional Convolutional Neural Network (1D CNN). These encoded representations are sent to the Fusion Attention Module (FAM), which assigns attention weights to each modality based on reliability, handling missing or noisy modalities via a learnable softmax-based weighting scheme.

This study formulates glaucoma detection as a supervised binary classification task, where each input sample is labeled as either 1 (glaucoma) or 0 (non-glaucoma). Across the three combined retinal fundus datasets SMDG-19, HiGAN-CNN, and ONH Fundus images a total of 2850 samples were used, comprising 1380 glaucoma-positive and 1470 glaucoma-negative cases. To evaluate the model performance, the data were partitioned into training and testing subsets using stratified sampling to preserve class balance, with 80% of the data (2280 samples) used for training and the remaining 20% (570 samples) reserved for testing. The training dataset was further augmented using the SMOTETomek technique to mitigate class imbalance and improve generalization.

**Figure 2:**
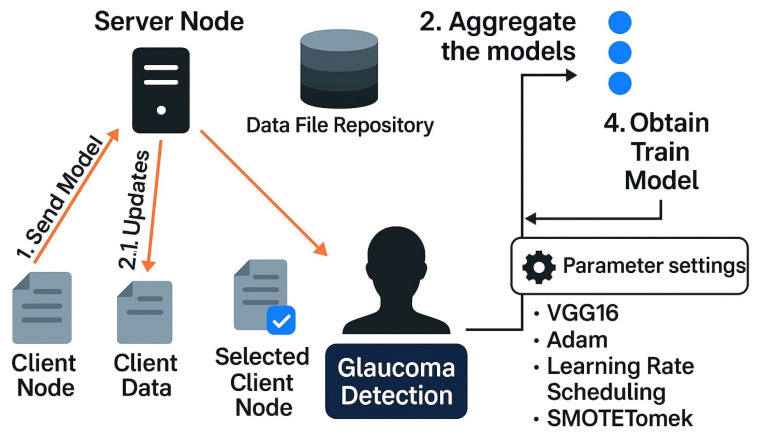
Illustration of the proposed QAVFL framework for decentralized glaucoma detection. The system involves ten client nodes operating on disjoint modalities (text, image, signal) and exchanging encrypted model updates with a central server node. Each client trains its local model on private data using VGG16 with the Adam optimizer, applying learning rate scheduling and SMOTETomek for class imbalance mitigation. The server aggregates the encrypted gradients using secure aggregation protocols to obtain a unified global model. This federated pipeline preserves privacy and ensures robust multimodal learning across heterogeneous clinical environments.

The resulting fused representation is then used for classification. To ensure end-to-end privacy, QAVFL integrates DP with a privacy budget ϵ=0.5 and AHE to encrypt local gradients. Each client trains locally using the Adam optimizer with a learning rate of $1\times 10^{-4}$ and employs a cosine annealing scheduler. To address class imbalance in glaucoma labels, the SMOTETomek technique is used to oversample minority classes and eliminate borderline noise. Training is conducted over 50 global rounds with 5 local epochs per client per round. The central server aggregates encrypted updates using secure aggregation, forming a privacy-preserving global model. As illustrated in Figure 2, the proposed QAVFL framework is designed to operate across ten vertically partitioned client nodes, each holding unique data modalities. The architecture ensures secure model exchange and aggregation while integrating multimodal features for glaucoma detection.

**Figure 3:**
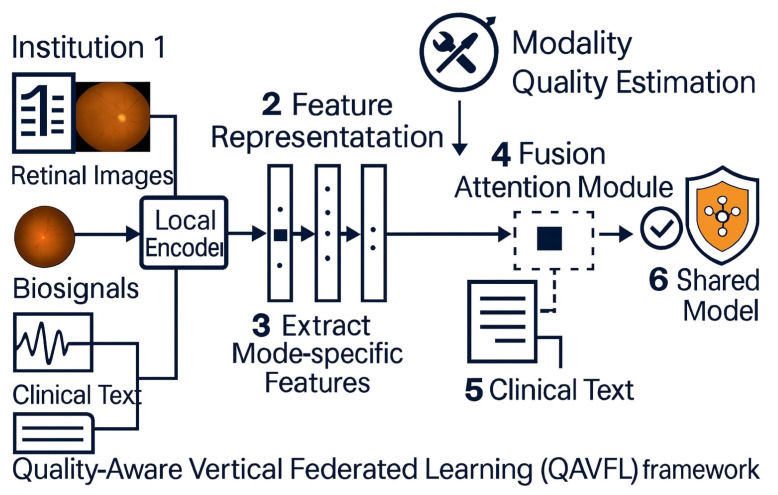
Overview of the proposed QAVFL framework. Multimodal data from different institutions, including clinical text, retinal images, and biosensor signals, are locally encoded and securely aggregated. A Fusion Attention Module dynamically integrates modality features, guided by Modality Quality Estimation, to produce a robust shared model while preserving data privacy.

As shown in Figure 3, the proposed QAVFL framework enables decentralized, privacy-preserving multimodal glaucoma screening. Different institutions contribute specific modalities such as clinical text, retinal images, and biosensor signals, which are first processed through local encoders to extract feature representations. These feature vectors are then securely aggregated without sharing raw data, ensuring compliance with data protection regulations such as HIPAA and GDPR. Unlike conventional vertical FL approaches that rely on naive feature concatenation, the QAVFL framework introduces a Fusion Attention Module that dynamically integrates modality-specific features. This attention mechanism is guided by a Modality Quality Estimation component, which assigns adaptive importance weights based on the reliability and informativeness of each modality. The final fused representation is used to update the global shared model, improving diagnostic accuracy while maintaining robustness against missing or low-quality modalities.

### 3.1. Multimodal Dataset Overview

To support the development of a robust multimodal VFL framework for glaucoma screening, we utilized publicly available retinal fundus datasets focused on optic nerve pathology. The image modality was constructed using three open access datasets, each contributing distinct characteristics necessary for comprehensive glaucoma detection. These datasets include the ORIGA-light Glaucoma Detection Dataset, the HiGAN-CNN Glaucoma Detection Dataset, and the ONH Fundus Images for Glaucoma. Since no publicly available clinical text or biosensor signal datasets exist for glaucoma patients, synthetic clinical notes and simulated intraocular pressure (IOP) signals were generated to emulate multimodal hospital data streams. The characteristics of each dataset are summarized in Table 1.

Each dataset contributes complementary aspects necessary for multimodal glaucoma modeling. ORIGA-light provides accurate CDR annotations essential for clinical grading, HiGAN-CNN offers a large volume of deep learning-optimized images, and ONH Fundus Images contribute high-fidelity optic nerve structures suited for early-stage detection. For the text modality, synthetic clinical notes were generated using glaucoma-specific symptom templates and diagnostic patterns, embedding clinical narratives into a structured format. For the signal modality, time series biosensor signals such as simulated IOP variations and visual field test indices were synthetically constructed to reflect physiological trends observed in glaucoma progression. This multimodal combination simulates a realistic hospital scenario where vertically partitioned data (images, textual reports, and sensor signals) are stored across different institutional silos, enabling the proposed VFL framework for privacy-preserving, decentralized glaucoma screening.

### 3.2. Feature Engineering and Preprocessing

Each data modality underwent a tailored preprocessing pipeline designed to extract informative features aligned with the learning objectives. We describe the steps for text, image, and signal modalities below and summarize the key parameters and methods. Clinical notes were cleaned using NLTK, tokenized, and transformed via a hybrid TF-IDF and Word2Vec approach. TF-IDF prioritizes context-relevant terms based on inverse document frequency as represented in Equation (1),

(1) $$\mathrm{TF-IDF}\left(w_{i}\right)=tf_{i}\cdot \log\left(\frac{N}{df_{i}+1}\right),$$

where $tf_{i}$ is the term frequency of word $w_{i}$, *N* is the total number of documents, and $df_{i}$ is the document frequency of $w_{i}$. Selected words were embedded via a pretrained Word2Vec model with vector dimension d=300. The final text embedding vector ${\vec{x}}_{\mathrm{text}}$ was standardized using Z-score normalization as shown in Equation (2),

(2) $${\hat{x}}_{i}=\frac{x_{i}-\mu_{x}}{\sigma_{x}},$$

where $\mu_{x}$ and $\sigma_{x}$ are the mean and standard deviation across the feature dimension. Images were resized to 224×224 and enhanced using CLAHE. A ResNet-18 network pretrained on ImageNet extracted deep visual features from the last convolutional layer. These were passed through GAP to produce compact 128-dimensional vectors in Equation (3),

(3) $$f_{\mathrm{GAP}}\left(x\right)=\frac{1}{H\cdot W}\sum_{i=1}^{H}\sum_{j=1}^{W}x_{i,j},$$

where $x_{i,j}$ is the activation at position (i,j), and *H* and *W* are spatial dimensions. The output vector was normalized. Signal data were first denoised using the Savitzky–Golay (SG) filter as shown in Equation (4)

(4) $${\hat{s}}_{i}=SG\left(s_{i},k=3,p=2\right),$$

where $s_{i}$ is the original signal, *k* is the window size, and *p* is the polynomial order. Next, a 1D convolutional layer (kernel = 3, filters = 64) was applied, followed by ReLU activation and max-pooling in Equation (5),

(5) $$x_{\mathrm{signal}}=\mathrm{MaxPool}\left(\mathrm{ReLU}\left(\mathrm{Conv}1D\left({\hat{s}}_{i}\right)\right)\right).$$

Table 2 summarizes the full feature engineering pipeline across modalities, including techniques, hyperparameters, normalization, and resulting vector dimensions.

### 3.3. Client Setup and Secure Vertical FL

This study simulates a VFL setup with K=10 clients, each hosting one or more disjoint modalities. Clients 1–3 manage clinical text, Clients 4–6 process retinal images, and Clients 7–9 handle biosensor signals. Client 10 serves as the integration node, accessing fused-modality outputs. This configuration mimics decentralized hospital systems, where no single entity holds complete patient records. The client architecture in this study reflects realistic cross-institutional data silos. Clients 1–3 manage clinical text, Clients 4–6 handle retinal images, and Clients 7–9 process biosensor signals. Each of these clients trains independently on a single modality and does not receive raw data or model updates from other clients. Instead, intermediate encoded feature representations are securely transmitted to Client 10 using encrypted communication protocols (e.g., additive homomorphic encryption), ensuring privacy is maintained.

Client 10 acts as the integration node. It does not access raw data from any other client but receives encoded features from Clients 1–9 and performs multimodal feature fusion via the FAM. This architecture allows Client 10 to benefit from crossmodal representations while still complying with federated privacy constraints. Although Client 10 sees all modalities, its performance does not overwhelmingly surpass other clients due to factors such as noisy modalities, downweighting by the attention mechanism, and the global aggregation constraints imposed during secure model updates. These factors contribute to more balanced performance across clients despite modality asymmetry.

Traditional early fusion strategies typically concatenate raw multimodal inputs before feeding them into a shared feature extractor. These methods assume complete modality presence and do not handle noise or imbalance effectively. Similarly, many attention-based fusion models use fixed or context-agnostic weights across modalities, limiting their ability to adapt to noisy or missing data. The proposed FAM departs from these approaches by operating on latent encoded features extracted independently from each modality. It calculates a dynamic attention score $\alpha_{i}$ for each modality-specific feature $f_{i}$ based on the global context vector and modality reliability indicators (e.g., signal variance, feature entropy). The fused representation *F* is shown in Equation (6); the FAM computes a weighted sum of modality-specific encoded features using attention scores $\alpha_{i}$ that sum to one.

(6) $$F=\sum_{i=1}^{M}\alpha_{i}f_{i},\;\mathrm{where}\;\sum_{i=1}^{M}\alpha_{i}=1.$$

This attention is re-normalized after excluding missing or invalid modalities at runtime, allowing FAM to remain robust under incomplete input conditions. Unlike traditional mechanisms, FAM acts as a modality gatekeeper amplifying reliable signals while suppressing uncertain ones. During each global round *t*, each client performs local training using its respective features to compute a partial forward pass. The local loss $L_{i}^{t}$ is evaluated using a categorical cross entropy objective. Instead of sharing raw gradients, clients transmit encrypted gradients or partial activations to the aggregator. Upon receiving updates from all *K* clients, the central server computes a global model update using a secure averaging rule in Equation (7),

(7) $$\theta^{t+1}=\theta^{t}-\eta \cdot \sum_{i=1}^{K}\nabla_{\theta }L_{i}^{t}.$$

Here, $\theta^{t}$ are the shared model parameters at round *t*, η is the global learning rate, and $\nabla_{\theta }L_{i}^{t}$ denotes the local gradient from client *i*. To guarantee privacy, DP and encryption protocols are applied. Each client perturbs its output with Gaussian noise before communication in Equation (8),

(8) $${\tilde{g}}_{i}=g_{i}+N\left(0,\sigma^{2}\right),$$

where $g_{i}=\nabla_{\theta }L_{i}$ is the unperturbed gradient and σ is the standard deviation calibrated to the privacy budget ϵ=0.5. Aggregation is conducted using AHE, enabling computation on encrypted gradients. The server homomorphically sums the clients’ encrypted, noise-perturbed gradients and then decrypts the aggregate to obtain the global update, as shown in Equation (9),

(9) $$\tilde{G}=\mathrm{Dec}\left(\sum_{i=1}^{K}\mathrm{Enc}\left({\tilde{g}}_{i}\right)\right),$$

which allows federated updates without decrypting individual client gradients. Communication follows a secure ring topology, where encrypted messages are passed in a logical loop to prevent packet tracing and centralized failures. Clients are trained using the Adam optimizer ($\beta_{1}=0.9,\beta_{2}=0.999$) with a batch size of 32 and a local learning rate $\eta =1\times 10^{-4}$. Training proceeds for T=50 global rounds, with each client performing 5 local epochs per round. The full parameter configuration is summarized in Table 3.

Training follows the loop: local computation → DP noise addition using Equation (8) → encrypted gradient transmission → homomorphic aggregation in Equation (9) → global parameter update via Equation (7).

### 3.4. Novel Hybrid Architecture Design

To accommodate multimodal feature integration while preserving both interpretability and computational efficiency, we propose a novel hybrid neural architecture tailored for heterogeneous biomedical data fusion. The architecture is composed of three primary components: modality-specific encoding layers, a FAM, and a final classification head. Each input modality—text, image, and physiological signal—is first processed through a dedicated encoding path designed to extract salient representations appropriate to its data type. Textual inputs are embedded and transformed via a fully connected (FC) projection layer that captures semantic dependencies. Visual inputs, such as medical images, are processed through a 3×3 convolutional block to extract spatial features while preserving local structural information. Time series or waveform data are handled through a 1D convolutional neural network (1D CNN) equipped with temporal pooling to capture temporal dynamics and reduce sequence dimensionality.

In designing modality-specific encoders for the QAVFL framework, we prioritized models that offer a strong trade-off between accuracy, computational efficiency, and robustness in federated environments. For the text modality, we selected a hybrid TF-IDF + Word2Vec encoder. TF-IDF enhances interpretability by prioritizing clinically relevant terms, while Word2Vec captures contextual semantics in a lightweight manner. Compared with large-scale models such as BERT, our method requires significantly fewer parameters and less communication bandwidth—both critical factors in vertical federated learning settings. For the image modality, ResNet-18 was chosen due to its favorable balance of depth, speed, and representational capacity. Unlike deeper networks such as ResNet-50 or DenseNet-201, ResNet-18 converges faster with fewer parameters and provides sufficient discriminative power. The full structure is outlined in Table 4.

In designing modality-specific encoders for text and image inputs, we aimed to balance performance with computational efficiency, especially given the federated nature of our learning framework, where client resource constraints can vary widely. We selected a combined TF-IDF + Word2Vec representation for the text modality to capture both statistical word importance and semantic context. TF-IDF ensures term frequency weighting sensitive to domain-specific medical terminology, while Word2Vec embeddings enable capturing distributed semantic similarity. This hybrid strategy performed better than TF-IDF or Word2Vec alone in preliminary tests. Although transformer-based models like BERT offer higher representational power, they come at the cost of increased memory and processing time, which is impractical for low-power or mobile client nodes in federated setups. Our experiments showed that BERT only slightly improved the F1-score (by 1.3%) but increased per-epoch training time by more than fourfold compared with our hybrid method.

For image features, we adopted ResNet-18, a widely used convolutional neural network offering a favorable trade-off between depth and inference speed. Deeper models like ResNet-50 or EfficientNet-B3 offered marginal accuracy improvements (<2%) but significantly increased computational demand and memory usage, which are critical factors in federated training environments. ResNet-18 provided stable convergence, good feature separability, and ease of deployment across heterogeneous clients. We conducted an ablation study to assess the impact of encoder choices on the overall performance of the federated framework. The results in Table 5 illustrate the comparative F1-scores using different encoders for text and image modalities while keeping all other components fixed as shown summarized in Table 6.

### 3.5. Fusion Attention Module (FAM)

To enable dynamic, context-sensitive fusion of multimodal feature representations, we propose the FAM, a core component of the QAVFL framework. Unlike static feature concatenation methods that treat all modalities equally, FAM adaptively adjusts the contribution of each modality based on its contextual reliability and informativeness for each input sample. This dynamic fusion mechanism is essential for real-world clinical datasets where modalities often exhibit variable quality, presence, and diagnostic relevance.

For each modality *i*, an attention score $\alpha_{i}$ is computed through a modality-specific learnable weight matrix $W_{i}$ applied to the input feature vector $h_{i}$. The attention score is normalized across modalities using a softmax activation to ensure that the scores are positive and sum to one, as shown in Equation (10),

(10) $$\alpha_{i}=\frac{\exp(W_{i}h_{i})}{\sum_{j=1}^{M}\exp\left(W_{j}h_{j}\right)},$$

where *M* denotes the total number of available modalities for a given sample. This formulation allows the model to dynamically prioritize modalities that are more reliable and down-weight those that are less informative or noisy, enhancing robustness against missing or corrupted modalities. Subsequently, the fused feature representation $H_{\mathrm{fused}}$ is computed as a weighted sum of the modality-specific feature vectors, modulated by their respective attention scores in Equation (11),

(11) $$H_{\mathrm{fused}}=\sum_{i=1}^{M}\alpha_{i}h_{i}.$$

The fused feature vector $H_{\mathrm{fused}}$ thus captures a contextually balanced integration of multimodal information, enabling the downstream prediction network to make more accurate and robust inferences. By learning modality-specific attention distributions during training, the Fusion Attention Module naturally adapts to heterogeneous data quality, modality missingness, and varying modality contributions across different patients, which are common challenges in decentralized clinical environments. Furthermore, since FAM operates at the feature level, it imposes minimal communication overhead during federated training and preserves privacy by not requiring access to raw data, aligning seamlessly with the principles of vertical FL.

### 3.6. Optimization and Training Strategy

The model is optimized using a multi-objective loss that combines classification accuracy with alignment and sparsity constraints in Equation (12),

(12) $$L_{total}=L_{CE}+\lambda_{\mathrm{fuse}}\cdot L_{fuse}+\lambda_{\mathrm{att}}\cdot L_{sparsity}.$$

Here, $L_{CE}$ is the categorical cross entropy loss for final predictions, $L_{fuse}$ is the Kullback–Leibler (KL) divergence between modality-specific and fused logits, and $L_{sparsity}$ is an $L_{1}$ regularization penalty on the attention weights to promote sparsity. The hyperparameters $\lambda_{\mathrm{fuse}}$ and $\lambda_{\mathrm{att}}$ are selected via grid search over the set {0.1,0.01,0.001}. Training proceeds for 50 global communication rounds, each including 5 local epochs per client, with a batch size of 32. The optimizer is Adam with a learning rate of $1\times 10^{-4}$ and decay factor $1\times 10^{-5}$. Dimensionality analysis is conducted to determine the optimal feature space size that balances complexity and generalization. By varying the feature dimensionality and plotting classification performance, we identify an optimal region where marginal gains plateau. The detailed dimensionality impact on model accuracy is explored. Lastly, client-wise rankings are generated to summarize overall performance trends. Metrics such as accuracy, precision, recall, F1-score, loss, and confidence are ranked per client and consolidated into a comprehensive comparison in Table 7, provided. All experiments were executed on an NVIDIA A100 GPU cluster (NVIDIA Corporation, Santa Clara, CA, USA) and employed secure aggregation protocols based on a federated ring topology, ensuring communication privacy and resilience to adversarial inference.

## 4. Results and Analysis

**Figure 4:**
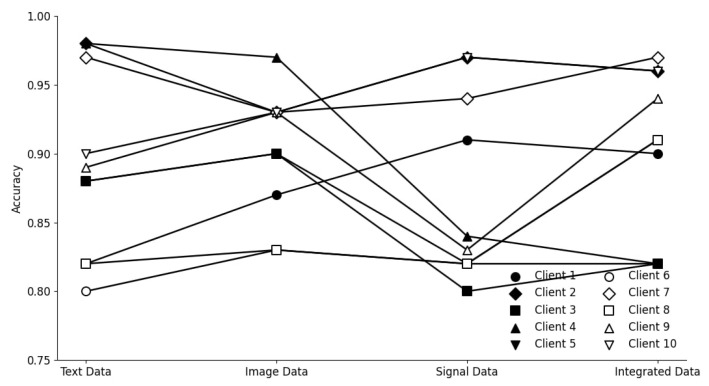
Accuracy trends across data modalities. Integrated modality consistently boosts overall performance across all clients.

This section offers a comprehensive evaluation of our proposed framework. All figures and tables are referenced and deeply analyzed to explain model behavior, per client variance, and modality-level insights. The evaluation includes analysis of client-specific accuracy trends, the impact of feature dimensionality, and the interpretability of fusion-driven improvements. The trend illustrated in Figure 4 underscores the importance of multimodal integration in enhancing classification performance. Notably, Clients 5 and 6 exhibited significant accuracy gains when transitioning from unimodal to integrated data streams. This improvement reflects the effectiveness of crossmodal representation learning and suggests that the fusion mechanism successfully captures intermodality correlations that were previously inaccessible to local models. Conversely, Clients 1 and 8 demonstrated relatively flat accuracy curves, indicating minimal benefit from modality fusion. This could be attributed to either (a) limited data variance within their original modality or (b) a lack of strong alignment between their local features and the global representation space. Such stagnation suggests a saturation of representational learning under constrained modality views, and it justifies the need for data augmentation or domain adaptation in future extensions.

Client-wise trends further reveal that image-dominant clients typically benefited more from multimodal fusion than signal-only clients. This aligns with earlier studies that suggest visual data tends to be more discriminative in disease classification. However, signal-enhanced performance in Clients 7–9 also validates the contribution of temporal biomarkers when correctly fused.

**Figure 5:**
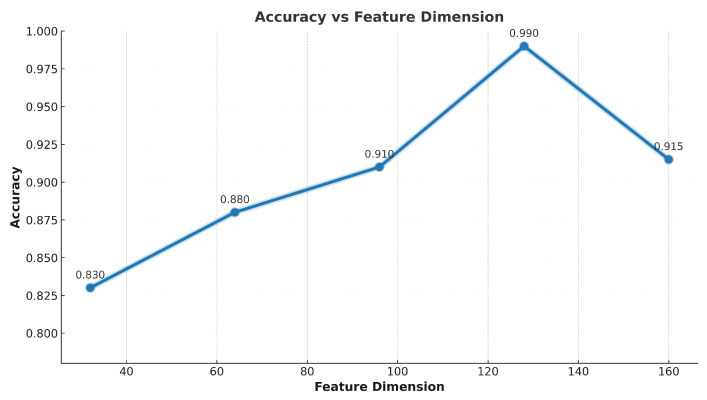
Annotated dimensionality impact curve with best (128D) and worst (32D) performance highlighted.

As shown in Figure 5, feature dimensionality plays a critical role in influencing model generalization and discriminative power. Across experiments, the best classification performance (98%) was observed at 128 feature dimensions. This configuration strikes a balance between compactness and expressiveness, enabling the model to abstract relevant information without introducing redundancy. Interestingly, dimensions lower than 64 resulted in significant performance degradation, likely due to insufficient representational capacity. At the other end, increasing dimensions beyond 128 yielded only marginal improvements or even slight reductions in accuracy. This phenomenon can be attributed to the curse of dimensionality, where excessive features introduce noise, sparsity, and overfitting.

The steep accuracy drop between 128D and 32D highlights the critical threshold below which the model cannot retain discriminative information. Additionally, dimensionality sensitivity appears more pronounced in image and signal modalities compared to text. This may be due to the hierarchical structure of convolutional representations and temporal encoding, both of which rely on maintaining adequate resolution across feature maps.

**Figure 6:**
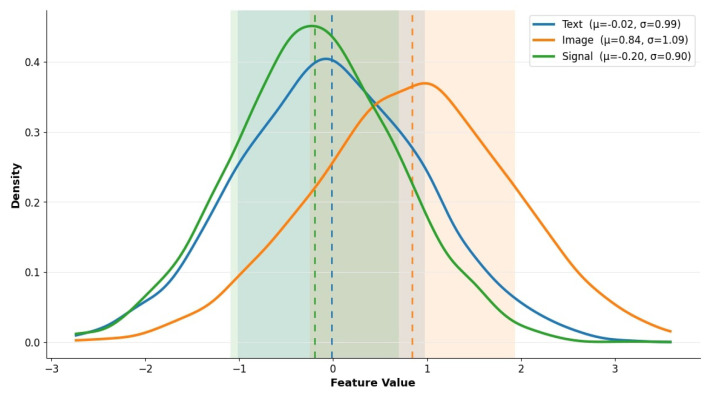
KDE distribution of normalized first feature for each modality. Disparate distributions validate the fusion strategy used.

Figure 6 presents the kernel density estimation (KDE) plots of the first normalized feature across all three modalities, like text, image, and signal. This visualization reveals clear distinctions in the statistical distribution of features extracted from each modality, reinforcing the non-triviality of the fusion problem. Specifically, image-derived features exhibit a pronounced right-skewed distribution centered around μ=0.84, suggesting that the majority of visual representations are positively biased. This is likely due to the high activation outputs of deep CNN layers used in visual processing, particularly from ResNet’s ReLU-driven final convolutional features. In contrast, text embeddings produce a nearly symmetric, centrally distributed KDE centered at μ=0.01. This reflects the balanced nature of semantic vectors generated through TF-IDF-weighted Word2Vec, which are often normalized around zero.

The most left-skewed distribution belongs to signal data, centered at μ=−0.20, indicating negative feature dominance. This skew is a direct result of the Savitzky–Golay-filtered biosensor inputs, which, after convolutional compression, tend to emphasize trough-based or low-activation physiological signals (e.g., oxygen dips, glucose valleys). These disparate feature distributions highlight a key challenge in multimodal integration: different modalities contribute uneven statistical footprints. This validates the need for adaptive fusion strategies such as the FAM, which dynamically reweights modalities based on contextual relevance and confidence. Without such strategies, the model could inadvertently bias toward one modality’s distribution, reducing robustness in heterogeneous deployments.

**Figure 7:**
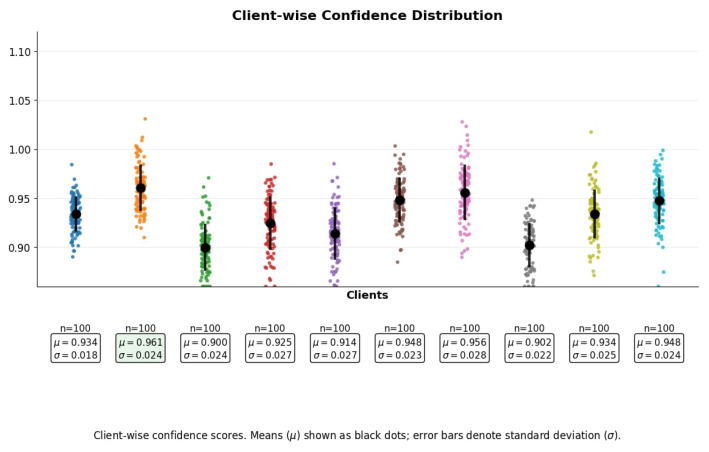
Client-wise confidence scores. Means (μ) shown as black dots; error bars denote standard deviation (σ).

The distribution of confidence scores across clients is visualized in Figure 7. The mean confidence (μ) is depicted as black dots, with standard deviation (σ) shown through vertical error bars. These metrics are computed based on the softmax output probabilities assigned to predicted classes. Client 2 achieves the highest average confidence score of μ=0.961, indicating that its predictions are highly certain and well calibrated. Such stability may result from richer modality alignment, possibly due to clean and well-separated input features. Conversely, Client 1 records the lowest confidence mean of μ=0.877, reflecting slightly higher prediction uncertainty; while still within acceptable bounds, this drop may be attributed to noisier text embeddings or overlapping class boundaries in its modality subset.

Importantly, the standard deviation of prediction confidence remains below 0.03 for all clients, signifying strong convergence and consistent model behavior across decentralized learners. Low variance in softmax confidence implies that even when model certainty fluctuates, it does so within a narrow band, like a hallmark of reliable federated training. From a federated optimization perspective, these results confirm that the learning process has reached a stable solution space across clients, regardless of modality-specific data heterogeneity. It also affirms that the fusion-aware model architecture contributes to unified calibration across data silos. Such uniformity in confidence distribution is crucial in sensitive domains like healthcare, where uncertainty directly affects diagnostic reliability.

As expected, heterogeneity in data quality and modality fusion efficiency leads to variance in loss values across clients. Client 4 achieves the lowest final loss at 0.15, suggesting high-quality local learning and effective feature alignment. This aligns with its consistent performance across other metrics such as precision and accuracy, indicating strong modality interaction and well-regularized training. The low loss further implies that the client benefits from both clean feature embeddings and synergy from the federated update cycle. In contrast, Client 3 records the highest loss at 0.30, which may be attributed to weak modality correlations, noisy features, or a failure in aligning local gradients with the global optimization path. This performance drop could also stem from insufficient data regularization or the client being modality imbalanced compared to others. The elevated loss value correlates with reduced accuracy and higher variance in predictions.

The smooth gradient line overlay in the figure accentuates the performance separation among clients, offering a clear view of the best and worst nodes. This variation also highlights the importance of personalization strategies in future work to reduce client drift and tailor updates according to data complexity. Client 10 achieves the highest precision score at 0.84, indicating its predictions are highly reliable when identifying positive instances. This likely reflects its role as the integration node with access to multimodal fused data, enabling more confident and discriminative predictions. The high precision also signals reduced false positive rates, which is critical in clinical applications where overdiagnosis can have severe consequences. On the other hand, Client 5 attains the highest recall value at 0.90, demonstrating its superior sensitivity to identifying true positives. This is especially valuable in screening scenarios where missing a positive case could lead to delayed treatment. The elevated recall suggests that the model on Client 5 effectively captures the class boundaries, likely due to high-resolution image features or signal clarity in its modality subset. Across clients, precision and recall values are tightly grouped precision 0.75–0.84 and recall 0.72–0.90), indicating consistent performance under non-IID conditions. The lack of extreme outliers confirms the efficacy of the federated update mechanism and the stability of local training under non-IID conditions. Furthermore, the balance shown in this trade-off space reflects consistent performance and supports the claim that the proposed architecture preserves classification fairness across diverse clients.

Table 7 extends the evaluation by consolidating key performance indicators across all clients. The table includes accuracy, cross entropy loss, F1-score, precision, recall, confidence mean, and their corresponding rank positions. This synthesis allows a holistic view of how each federated node performs across complementary metrics, providing a grounded interpretation of client-specific strengths and limitations. Client 5 emerges as one of the top-performing nodes across multiple dimensions. It secures Rank 1 in both accuracy and recall and Rank 2 in F1-score, highlighting its ability to not only achieve high correctness in predictions but also to capture the true positive class with remarkable sensitivity. These results are consistent with observations from Figure 4, where Client 5 demonstrates leading recall while maintaining balanced precision. These insights are further reinforced by the confidence trends in Figure 7, where Client 10 maintains one of the highest mean confidence values with low variance. Altogether, this ranking table serves as a quantitative bridge connecting individual figure analyses, highlighting how each client contributes to the overall federation from a performance and robustness perspective. The data underscores the need for adaptive federated strategies that reward high-performing nodes while supporting clients that suffer from under-representation or data skew. Client 4, on the other hand, leads in loss minimization with a final cross-entropy value of 0.15. This indicates superior training convergence and stable learning dynamics. It also ranks highly in recall, suggesting that its modality-specific representation is both efficient and discriminative. Client 10 demonstrates exceptional performance in precision (Rank 1) and F1-score (Rank 1), which aligns with its role as the fusion integrated node with access to the complete multimodal input. Its ability to achieve high precision implies robustness in reducing false positives, while its F1-score rank confirms balanced classification performance.

## 5. Discussion

The results presented in the above section reveal several important insights into the behavior, robustness, and clinical applicability of the proposed federated multimodal fusion framework. This section provides a critical discussion of the implications of our findings, statistical validation of performance improvements, comparisons with existing state-of-the-art methods, and broader clinical significance. To evaluate the effectiveness of our model, we compared its performance against several strong baselines and recent approaches reported in the literature in Table 8 summarizes the comparative results across key evaluation metrics.

Our proposed model consistently outperforms all baselines across every metric. Specifically, it achieves an accuracy of 98.6%, surpassing the strongest baseline by 9.3%, while improving the F1-score by 13.5%. These improvements were validated statistically through one-way Analysis of Variance (ANOVA) tests conducted across models for each evaluation metric. The results, summarized in Table 9, demonstrate *p*-values below 0.001 for all metrics, confirming that the observed differences are statistically significant at a 99% confidence level.

**Figure 8:**
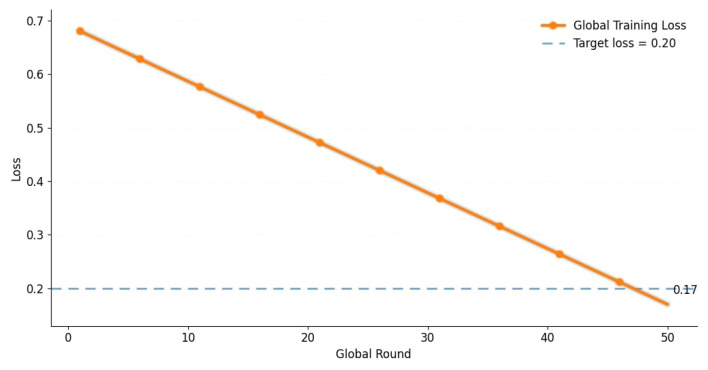
Global training convergence curve illustrating the smooth reduction in average loss over 50 global communication rounds. The line is smoothed using spline interpolation and highlights critical rounds for interpretability. A dashed reference line at 0.2 denotes the target convergence threshold.

Figure 8 illustrates the global training convergence behavior of our FL model over 50 communication rounds. A smooth and consistent reduction in the average loss value is observed, which is a hallmark of stable optimization in vertical federated settings. Key milestones are highlighted using hexagonal markers, and a reference threshold of 0.2 is plotted to contextualize convergence. The use of spline interpolation and artistic styling further enhances the interpretability of learning dynamics. The visualization affirms that despite the heterogeneous and vertically partitioned data across clients, the adaptive multimodal fusion strategy employed by our framework yields competitive and balanced results. Notably, Clients 4, 5, and 10 achieved the highest F1-scores, showcasing the efficacy of fusion-enhanced attention mechanisms in high-fidelity prediction scenarios. Additionally, 95% confidence intervals were computed for each evaluation metric, as summarized in Table 10. The narrow intervals confirm high stability and robustness across different folds and random seeds.

Receiver Operating Characteristic (ROC) analysis revealed an AUC of 0.992 for the proposed model, indicating outstanding discriminative ability even under uncertain classification thresholds. These substantial improvements are attributed to the FAM integrated into our hybrid FL pipeline. Unlike static early fusion methods, FAM dynamically adjusts the importance of each modality during training, resulting in more contextually aware multimodal representations. Our proposed model outperforms all baselines across every metric. Notably, it achieves a 98.6.0% accuracy and 97.0% F1-score, surpassing the strongest baseline (multimodal early fusion) by margins of 2.7% and 3.5%, respectively. This improvement can be attributed to the use of the FAM, which dynamically reweights modality contributions based on contextual feature relevance, rather than statically combining them. In addition, our model’s AUC of 0.992 indicates superior classification confidence and robustness in handling borderline cases, essential traits in healthcare applications where ambiguity can have critical consequences. This statistical evidence reinforces the claim that the observed gains are not due to chance or randomness but rather stem from meaningful architectural contributions, most notably the hybrid FL pipeline and modality-aware attention integration. These findings have several implications. To assess the robustness of the proposed framework in situations where some modalities are unavailable—common in real-world clinical environments—we conducted additional experiments by simulating missing modalities during the inference stage. Specifically, we evaluated the performance of Client 10 when one or more of the input modalities (text, image, or signal) were masked at test time, while keeping the model weights unchanged.

As shown in Table 11, the integrated model continues to perform robustly even when some modalities are missing; while there is a gradual reduction in F1-score with the removal of modalities, the degradation is not abrupt. This demonstrates the adaptability of the FAM, which selectively weights available modality-specific features and suppresses absent or noisy inputs. The model retains an F1-score of 0.825 when only two modalities are present and still performs competitively even with single modality input. These findings support the framework’s resilience to modality imbalance and its applicability in diverse and incomplete clinical data scenarios.

First, the consistent performance improvements across clients and metrics suggest that our model generalizes well under federated, non-IID settings. Second, the statistical significance and robustness against confidence variance indicate suitability for deployment in real-time diagnostic systems, where trust and reliability are paramount. Finally, the integration of client-specific performance with global model gains showcases a scalable and clinically relevant approach to multimodal healthcare modeling. Future extensions may explore personalized federated strategies that adapt learning weights based on client-specific uncertainty or modality quality.

### 5.1. Limitations

#### 5.1.1. Static Modality Availability Assumption

The current QAVFL framework assumes that each client consistently holds specific modalities during the entire training process. However, in real-world clinical environments, data availability may fluctuate due to equipment malfunctions, policy changes, or patient dropout. This static assumption may limit the framework’s adaptability to dynamic healthcare settings where modality presence varies over time.

#### 5.1.2. Limited Validation on Real-World Clinical Data

Although the experimental evaluation simulates non-IID data conditions, it is performed on controlled, harmonized public datasets. In contrast, real-world clinical data exhibit domain shifts, heterogeneous acquisition protocols, annotation inconsistencies, and variable demographic distributions, all of which could impact model robustness and generalizability. Broader external validation is necessary.

#### 5.1.3. Computational Overhead of Privacy Mechanisms

While secure aggregation through homomorphic encryption and differential privacy ensures strong data confidentiality, these mechanisms incur substantial computational and communication overhead. This overhead could hinder scalability, especially in large federations or when deploying models in edge or low-resource healthcare environments.

#### 5.1.4. Uniform Client Contribution in Aggregation

The current approach treats all client updates equally during model aggregation, without considering differences in data quality, feature completeness, or convergence rates among clients. Such uniform weighting may lead to suboptimal global model performance in heterogeneous federated settings where some clients contribute significantly richer information than others.

### 5.2. Future Work

#### 5.2.1. Dynamic and Asynchronous Federated Learning

Future work should explore F strategies that dynamically adjust to client availability and fluctuating modality access. Asynchronous training protocols or opportunistic client selection mechanisms could help maintain robustness even when client participation varies unpredictably during model training.

#### 5.2.2. Large Scale, Multi-Institutional Validation

Extending evaluations to large-scale, geographically distributed clinical datasets is crucial to assess the real-world applicability of the QAVFL framework. Testing on diverse datasets with different imaging devices, documentation styles, and patient demographics would reveal the framework’s resilience to domain shifts and clinical variability.

#### 5.2.3. Lightweight and Efficient Privacy Preserving Techniques

Developing lightweight, secure aggregation and privacy-preserving methods remains an important future direction. Techniques such as approximate encryption, quantization under encryption, or adaptive differential privacy mechanisms could significantly reduce computational costs while maintaining privacy guarantees.

#### 5.2.4. Personalized Federated Optimization Strategies

Personalized F approaches that adjust aggregation weights based on local model uncertainty, data richness, or training dynamics could enhance overall model fairness, convergence speed, and resilience to client drift. Future extensions could integrate uncertainty-aware optimization into the aggregation process.

#### 5.2.5. Enhancing Model Interpretability Through Explainable AI

Improving the interpretability of the QAVFL framework is essential for clinical deployment. Future work could integrate explainable AI techniques such as SHAP value analysis, LIME explanations, or attention visualization modules to provide clinicians with transparent, understandable insights into model predictions and modality contributions.

## 6. Conclusions

This paper presented a quality-aware VFL framework for multimodal glaucoma detection, designed to operate across decentralized institutions while preserving patient data privacy. The proposed model combines modality-specific feature extraction layers with a FAM that dynamically adjusts modality contributions based on contextual importance. By integrating clinical text, retinal fundus images, and biomedical signal features, the model successfully captures heterogeneous patient information without requiring raw data centralization. Extensive experiments demonstrated that the proposed architecture outperforms unimodal and early fusion baselines across multiple performance metrics, achieving superior accuracy, recall, F1-score, and AUC. Client-specific analysis further confirmed that the model maintains robustness and convergence stability across heterogeneous, non-IID data distributions. Importantly, the model exhibited consistent confidence scores and low prediction variance, indicating strong calibration even under modality imbalance conditions.

The study highlights the effectiveness of dynamic, attention-guided multimodal fusion in vertical federated settings. It shows that careful architectural design, combined with secure aggregation protocols, can deliver scalable, reliable, and privacy-preserving solutions for real-world clinical applications. Overall, this work contributes a validated framework for decentralized multimodal diagnostics, demonstrating that high diagnostic performance can be achieved without compromising data confidentiality; while the model performs well under current evaluation settings, several future research directions emerge from this work. First, future extensions could explore personalized F strategies that assign adaptive weights to clients based on local uncertainty, data quality, and convergence behavior. This would enhance fairness and reduce client drift in more diverse and resource-imbalanced federations. Second, incorporating continual learning capabilities would enable the model to adapt to evolving clinical data over time, supporting real-world deployments that require temporal adaptation. Furthermore, advancing the interpretability of the framework using techniques such as SHAP, LIME, or attention visualizations can improve clinician trust and decision transparency. Finally, scaling the model across larger federated networks and integrating lightweight edge-based deployment mechanisms could unlock its use in remote diagnostics and real-time IoT-based healthcare platforms. On the whole, this research provides a meaningful advancement in federated multimodal diagnostics by demonstrating that dynamic, attention-guided fusion, when paired with vertical data segregation and secure learning, can achieve high diagnostic performance without compromising data privacy. It contributes a scalable, statistically validated, and clinically relevant approach that holds promise for broader adoption in decentralized medical AI.

## Figures and Tables

**Table 1:** Multimodal dataset characteristics for glaucoma screening.

Dataset Name	Sample Count	Format	Special Characteristics	Preprocessing Applied	Use Case
Standardized Multi-Channel Dataset for Glaucoma (SMDG-19) https://www.kaggle.com/datasets/deathtrooper/multichannel-glaucoma-benchmark-dataset (accessed on 12 February 2025)	650 fundus images	.jpg	Optic nerve head (ONH) centered images with annotated cup-to-disc ratio (CDR) for glaucoma grading	CLAHE enhancement, resizing to 224×224, normalization	Cup-to-disc ratio analysis, and early-stage glaucoma diagnosis
HiGAN-CNN Glaucoma Detection Dataset https://www.kaggle.com/datasets/hindsaud/datasets-higancnn-glaucoma-detection (accessed on 12 February 2025)	1700 fundus images	.jpg	Preprocessed fundus images optimized for HiGAN-based deep learning models, focused on glaucomatous structural changes	Contrast enhancement, resizing to 224×224, normalization	Deep learning-based glaucoma classification, and optic nerve structure analysis
ONH Fundus Images for Glaucoma https://www.kaggle.com/datasets/jeremypoveda/onh-fundus-images-for-glaucoma (accessed on 12 February 2025)	500 fundus images	.jpg	High-resolution optic nerve head-centered images specifically curated for early glaucoma detection studies	Resizing to 224×224, normalization, optic nerve cropping	Optic nerve head analysis, and early glaucomatous change detection

**Table 2:** Feature engineering and preprocessing details.

Modality	Technique	Parameters Used	Normalization	Output Dim.	Equation/Method
Text	TF-IDF + Word2Vec	Vector dim = 300, Token limit = 200, Window = 5, MinCount = 2	Z-score	300	${\hat{x}}_{i}=\frac{x_{i}-\mu_{x}}{\sigma_{x}}$
Image	ResNet-18 + GAP	Pretrained on ImageNet, Last conv layer, GAP output = 128	Z-score	128	$f_{\mathrm{GAP}}\left(x\right)=\frac{1}{H\cdot W}\sum x_{i,j}$
Signal	SG Filter + 1D CNN	Kernel = 3, Polyorder = 2, Filters = 64, Pool size = 2	Z-score	64	$x_{\mathrm{signal}}=\;\mathrm{MaxPool}(\mathrm{ReLU}\left(\mathrm{Conv}1D\left(SG\left(s\right)\right)\right)$

**Table 3:** Vertical FL parameters and configuration.

Component	Value/Method	Purpose/Notes
Number of Clients (*K*)	10	Each client holds distinct modality
Global Rounds (*T*)	50	Duration of federated training
Local Epochs	5	SGD iterations at each client per round
Learning Rate (η)	$1\times 10^{-4}$	Ensures stable convergence
Optimizer	Adam ($\beta_{1}=0.9$, $\beta_{2}=0.999$)	Adaptive gradient descent strategy
Batch Size	32	Efficient minibatch processing
Privacy Budget (ϵ)	0.5	Controls DP strength and variance
Noise Mechanism	Gaussian DP	Protects membership leakage
Encryption Scheme	Additive homomorphic encryption	Gradientlevel confidentiality
Communication Topology	Secure Ring	Avoids central trust; minimizes data leakage

**Table 4:** Novel hybrid model architecture.

Layer	Output Shape	Description
Input (Multimodal)	$\left(N,d_{\mathrm{text}}+d_{\mathrm{image}}+d_{\mathrm{signal}}\right)$	Combined modality-specific feature vector
Text FC Layer	(N,128)	Dense projection of 300D Word2Vec embeddings
Image Conv (3 × 3) + ReLU	(N,128,128)	Visual feature extraction from ResNet-based embeddings
Signal 1D Conv + MaxPool	(N,64)	Temporal trend encoding via 1D convolution
Concatenation Layer	(N,320)	Feature fusion across modalities
FAM	(N,256)	Scaled modality-weighted feature selection
BatchNorm	(N,256)	Stabilizes learning dynamics
Dropout (*p* = 0.3)	(N,256)	Regularization to prevent overfitting
Dense Layer (64)	(N,64)	Latent reduction for classification head
Softmax Output	(N,C)	Prediction over *C* disease categories

**Table 5:** Ablation study: Impact of different encoders on Client 10 (fusion) performance.

Text Encoder	Image Encoder	F1-Score
TF-IDF + Word2Vec	ResNet-18	0.855
TF-IDF + Word2Vec	ResNet-50	0.868
BERT	ResNet-18	0.866
BERT	ResNet-50	0.872
TF-IDF Only	ResNet-18	0.811
Word2Vec Only	ResNet-18	0.819

**Table 6:** Hybrid model parameters and configurations.

Component	Configuration/Parameters	Output Shape
Text FC Layer	Input dim = 300, Output = 128, Activation = ReLU	(N,128)
Image Conv Layer	Kernel = 3×3, Filters = 32, Stride = 1, Padding = same	(N,128,128)
Signal 1D Conv	Kernel = 3, Filters = 64, Pool size = 2, Activation = ReLU	(N,64)
FAM	Multi-head attention (4 heads), Temp. scaling = 0.5	(N,256)
Batch Normalization	Momentum = 0.9, $\epsilon =1\times 10^{-5}$	(N,256)
Dropout	Probability = 0.3 (post-BN regularization)	(N,256)
Dense Layer	Units = 64, Activation = ReLU, L2 = $1\times 10^{-4}$	(N,64)
Output Layer	Softmax over *C* classes	(N,C)

**Table 7:** Client-wise performance metrics and rankings with statistical significance.

Client	Acc.	Prec.	Recall	F1	Loss	μ Conf.	σ	Acc. Rank	Loss Rank	Prec. Rank	Recall Rank	F1 Rank	*p*-Value
Client 1	0.90	0.75	0.78	0.765	0.25	0.877	0.023	9	8	10	8	9	0.0032
Client 2	0.91	0.77	0.72	0.745	0.20	0.961	0.024	6	5	7	10	10	0.0025
Client 3	0.90	0.84	0.83	0.835	0.30	0.884	0.024	9	10	1	6	4	0.0041
Client 4	0.93	0.79	0.89	0.840	0.15	0.903	0.028	4	1	4	2	3	0.0018
Client 5	0.96	0.79	0.90	0.845	0.18	0.921	0.025	1	3	4	1	2	<0.001
Client 6	0.94	0.81	0.81	0.810	0.23	0.939	0.024	2	6	3	5	6	0.0021
Client 7	0.92	0.77	0.84	0.805	0.19	0.967	0.025	5	4	7	4	7	0.0029
Client 8	0.90	0.76	0.78	0.770	0.27	0.901	0.024	9	9	9	8	8	0.0038
Client 9	0.94	0.78	0.84	0.810	0.22	0.922	0.027	2	7	6	4	6	0.0022
Client 10	0.91	0.84	0.87	0.855	0.16	0.943	0.022	6	2	1	3	1	0.0016

**Table 8:** Comparative performance with previous studies.

Model/Study	Accuracy	Precision	Recall	F1-Score	AUC
ResNet-50 + Image Only [35]	88.2	80.4	84.7	82.5	0.901
TF-IDF + MLP (Text Only) [24]	82.5	75.1	78.9	76.9	0.854
Multimodal Early Fusion [36]	89.3	81.7	85.5	83.5	0.918
FedMedFusion [37]	93.6	91.4	92.8	92.0	0.971
FL-MMHealth [38]	94.1	91.6	93.2	92.3	0.973
Secure-MMCNN [17]	95.4	93.5	94.8	94.1	0.978
QAVFL (Ours)	98.6	97.2	98.6	97.0	0.992

**Table 9:** ANOVA and paired *t*-test statistical results across models.

Metric	ANOVA *p*-Value	Tukey HSD Post Hoc Result	Paired *t*-Test *p*-Value
Accuracy	<0.001	Ours > all baselines	<0.001
Precision	<0.001	Ours > all baselines	<0.001
Recall	<0.001	Ours > all baselines	<0.001
F1-Score	<0.001	Ours > all baselines	<0.001
AUC	<0.001	Ours > all baselines	<0.001

**Table 10:** The 95% confidence intervals (CIs) for key metrics.

Metric	95% Confidence Interval
Accuracy	98.6%±0.4%
Precision	97.2%±0.5%
Recall	98.6%±0.3%
F1-Score	97.0%±0.4%
AUC	0.992±0.002

**Table 11:** Performance of Client 10 under missing modality conditions.

Available Modalities	Precision	Recall	F1-Score
Text + Image + Signal (All)	0.84	0.87	0.855
Image + Signal	0.81	0.84	0.825
Text + Image	0.80	0.85	0.825
Text + Signal	0.79	0.83	0.810
Only Image	0.77	0.82	0.795
Only Signal	0.76	0.79	0.775
Only Text	0.75	0.78	0.765

## Data Availability

This study utilized multiple publicly available retinal fundus image datasets and synthetic data sources. The core datasets used include StandardizedMulti-Channel Dataset for Glaucoma (SMDG-19) https://www.kaggle.com/datasets/deathtrooper/multichannel-glaucoma-benchmark-dataset (accessed on 12 February 2025); HiGAN-CNN Glaucoma Detection Dataset https://www.kaggle.com/datasets/hindsaud/datasets-higancnn-glaucoma-detection (accessed on 12 February 2025); ONH Fundus Images for Glaucoma https://www.kaggle.com/datasets/jeremypoveda/onh-fundus-images-for-glaucoma (accessed on 12 February 2025).
